# Thioxanthone Skeleton-Based One-Component Macro-Photoinitiator Reduces Oxygen Inhibition and Migration Through Cooperative Effect

**DOI:** 10.3390/polym17162252

**Published:** 2025-08-20

**Authors:** Yiyun Du, Jingyan Zhang, Tianyi Han, Yi Zhu

**Affiliations:** 1 International Research Center for Photoresponsive Molecules and Materials, Jiangnan University, Wuxi 214122, China; 6240609001@stu.jiangnan.edu.cn (Y.D.); 6210612049@stu.jiangnan.edu.cn (J.Z.); 2 State Key Laboratory of Tribology in Advanced Equipment, Tsinghua University, Beijing 100084, China; 3 Key Laboratory of Synthetic and Biological Colloids, Ministry of Education, School of Chemical and Material Engineering, Jiangnan University, Wuxi 214122, China

**Keywords:** photocuring, photoinitiator, oxygen inhibition, migration, fluorinated molecules

## Abstract

The oxygen inhibition and migration of micromolecules which stem from photoinitiators (PIs) remain two critical challenges to address in radical photocuring. In this work, we reported a one-step ternary copolymerization strategy to construct a one-component macromolecular photoinitiator (PPI) using polymerizable thioxanthone (TX), amine (N), and fluorinated alkane (F) as monomers. Then, we utilize the low surface energy of F unit and macromolecular skeleton to reduce oxygen inhibition and migration. Compared to micromolecule TX, PPI also exhibits a broad absorption in the 250–430 nm range, and a higher molar extinction coefficient. The effects of the TX, N, and F component ratios on the photoinitiation efficiency of PPI were systematically investigated, and the photopolymerization kinetics revealed that the increased content of F unit can eliminate the oxygen inhibition of PPI. As a result, PPI demonstrates the more superior photoinitiation efficiency compared to the traditional TX/N two-component macromolecule photoinitiation system. Migration experiments indicated that there is a 60% reduction in the migration rate for PPI compared to the TX/N photoinitiation system. This work provides an effective strategy to address oxygen inhibition and micromolecule migration issues in radical photocuring, showing potential applications in food and pharmaceutical packaging fields.

## 1. Introduction

Photocuring is an emerging strategy for manufacturing high-performance materials, offering distinct advantages [1,2,3,4,5], including precise spatiotemporal control, cost-effectiveness, and environmental friendliness, with current extensive applications in advanced manufacturing fields including printing inks [6,7], food-grade packaging [8], and microelectronic photolithography [9,10]. As the core component to initiate photopolymerization, photoinitiators (PIs) occupy an irreplaceable role in the photocuring formulations. However, during the photocuring process, oxygen can react with free radicals, resulting in chain transfer/termination, inhibiting radical polymerization, and causing incomplete curing, surface tackiness, and other problems, which will affect the properties of materials [11]. In addition, photocurable materials migrate toxic micromolecules, such as micromolecule PIs and the cracking products of PIs, thus limiting the application of photocuring in food-grade packaging and other fields [12,13]. Both oxygen inhibition and micromolecules migration issues originate from the inherent characteristics of the photocuring system. Therefore, the development of one-component PIs with the reduction of oxygen inhibition and migration has significant research value and challenges.

The selection and construction of a chromophore skeleton is the most essential component in the design of PIs. Among numerous chromophores, thioxanthone (TX) has good absorption in the ultraviolet-visible (UV-Vis) spectrum; it can match Light-Emitting Diode (LED), and has other advantages, including a long triplet lifetime and high quantum yield [14]. Therefore, TX has been widely applied in photopolymerization and photocatalytic fields [15,16,17]. To the best of our knowledge, PIs based on the TX skeleton are mainly used as Norrish II type PIs [17], such as the commercial photoinitiator 2-isopropylthioxanthone (ITX), which has good application in actual production. However, Norrish II PIs require hydrogen transfer with hydrogen donors to generate carbon-centered radicals for initiating polymerization [18]. With the progression of polymerization, the increased system viscosity hinders hydrogen transfer reaction between the PIs and hydrogen donors, resulting in a decrease in initiation efficiency. Moreover, the addition of hydrogen donors such as amines may introduce issues that affect material properties, including odor, yellowing, poor compatibility, toxicity, and increased migration [19]. Like other micromolecule photoinitiation systems, oxygen inhibition and micromolecule migration have also limited the development of a two-component photoinitiation system based on a TX backbone [20].

Currently, the primary strategy to address the migration issue is by incorporating TX and its derivatives with hydrogen donors into macromolecular architectures, which limit the migration of micromolecules after photolysis and increase the hydrogen transfer efficiency between TX derivatives and hydrogen donors, thus improving the initiation efficiency. The researchers synthesized polymers or hyperbranched macromolecules with active groups on the side chains, and then grafted TX derivatives to the side chains [21,22,23]. Polymerizable TX derivatives and hydrogen donors can also be utilized to construct one-component macromolecular PIs through copolymerization [24,25,26,27]. Furthermore, the current strategy to solve the oxygen inhibition in radical photocuring from the perspective of PIs is to incorporate fluorinated or siloxane-containing segments into the photoinitiator skeleton to design self-floating PIs; additionally, functionalized PIs can migrate to the top layer, and thus the high concentration of PIs on the surface initiates the polymerization of monomers and forms a cured film, which acts as a physical barrier to prevent oxygen from diffusing into the system [28,29]. Other researchers grafted TX to polyhedral oligomeric silsesquioxane (POSS). Thiols on POSS can react with acrylate monomers to form thiol-ene polymer on the surface layer, which acts as a physical barrier to prevent oxygen from diffusing into the system [30]. However, there are no reports of unimolecular PIs based on TX skeleton that can address both the oxygen inhibition and micromolecule migration issues simultaneously.

Therefore, this study reported a one-component PPI, which is based on the TX skeleton, and utilized polymerizable thioxanthone (TX), amine (N), and fluorine-containing alkane (F) as monomers copolymerized on a macromolecular chain via a one-step ternary copolymerization strategy. We applied it to free radical photocuring and systematically studied the effect of the proportion of each component unit on the photopolymerization kinetics, and its reduction of oxygen inhibition and migration behavior were further explored. This work presents a cooperative effect strategy utilizing the low surface energy of F unit and macromolecular skeleton to address the persistent challenges of oxygen inhibition and micromolecule PIs migration in free radical photocuring. Furthermore, the reactivity of N unit was used to improve photoinitiation efficiency, demonstrating promising potential for applications in food-grade packaging and biomedical fields.

## 2. Materials and Methods

### 2.1. Material

The experimental materials used in this paper are shown in Table 1.

### 2.2. General Instruments

^1^H NMR were obtained using a Bruker AVANCE III HD 400 MHz nuclear magnetic resonance spectrometer, Switzerland, Germany. UV-Vis absorption spectra and steady-state photolysis spectra were obtained using a Shimadzu UV-1990i UV-Vis spectrophotometer, Kyoto, Japan. Real-time infrared (RT-IR) was tested using a Thermo Fisher Nicolet 6700 total reflection Fourier infrared spectrometer, Waltham, MA, USA. Electron spin resonance (ESR) was obtained using an EMXplus-10/12 electron spin resonance spectrometer, Switzerland, Germany. Number-average molecular weights were obtained using an EcoSEC HLC-8320 gel permeation chromatograph (GPC) from Tosoh Corporation, Japan. The model number of light sources used is Lumen Dynamics OmniCure S1000, Mississauga, Canada.

### 2.3. Synthesis

**Scheme 1:**
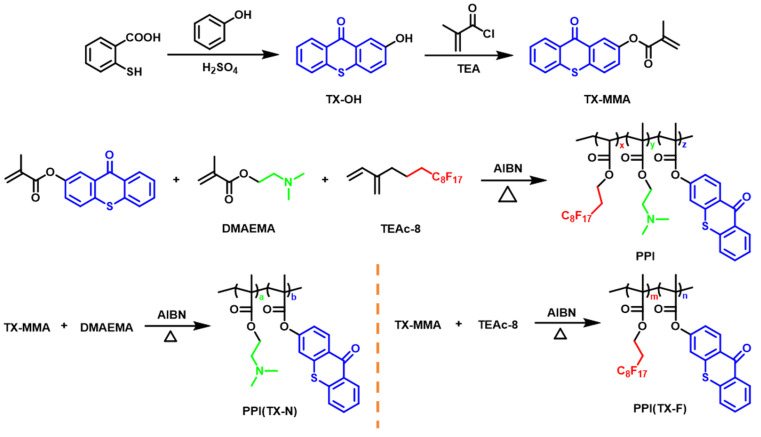
Synthetic routes of PPI.

The general procedure for the synthesis of PPI is summarized in Scheme 1. ^1^H NMR and GPC spectra of intermediate products are given in the Supplementary Materials (Figures S1–S4). 2-Hdroxythioxanthone (TX-OH) was synthesized by condensation cyclization of phenol and thiosalicylic acid catalyzed by concentrated sulfuric acid. TX-OH was alcoholized with methacryloyl chloride to generate methacrylate-2-thioxanthone ester (TX-MMA). Then it was thermally polymerized with N and F unit in the presence of thermal initiator AIBN. Four types of ternary macromolecular PIs were designed and synthesized by changing the ratio of PIs, polymerization time, and content of the thermal initiator; binary macromolecular PIs were synthesized for comparative study. All the reactions were carried out in a dark environment.

Synthesis of TX-OH: Thiosalicylic acid (3.08 g, 0.02 mol) was slowly added to concentrated sulfuric acid (40 mL). After complete dissolution, phenol (5.64 g, 0.06 mol) was added, stirred at room temperature for 2 h, then heated to 75 °C and stirred for 4 h. After heating, it was cooled to room temperature and stirred overnight. At the end of the reaction, the reaction mixture was added to 400 mL boiling water, the precipitate was filtered, then washed with water, collected, and dried in a vacuum oven at 60 °C for 12 h, and then recrystallized from dioxane/water to produce a yellow powder in a yield of 67.4%. ^1^H NMR (400 MHz, DMSO-*d*_6_) δ 10.15 (s, 1H), 8.47 (d, *J* = 8.0 Hz, 1H), 7.88 (d, *J* = 2.8 Hz, 1H), 7.81 (d, *J* = 8.1 Hz, 1H), 7.78–7.71 (m, 1H), 7.69 (dd, *J* = 8.7, 1.2 Hz, 1H), 7.56 (td, *J* = 7.6, 7.0, 1.4 Hz, 1H), 7.29 (dd, *J* = 8.7, 2.8 Hz, 1H).

Synthesis of TX-MMA: In an oven-dried round-bottom flask, TX-OH (1.13 g, 0.05 mol) was added to dry DCM (20 mL) and triethylamine (1.39 mL, 0.01 mol) was added and stirred at 0 °C for 15 min under a nitrogen atmosphere. Methacryloyl chloride (0.968 mL, 0.01 mol) was added dropwise to the reaction solution and stirred on an ice bath. After completion of the dropwise addition, the reaction was allowed to warm naturally to room temperature and react overnight. After completion of the reaction, the precipitate of triethylammonium chloride was removed by filtration, and the organic phase (DCM) was washed with acidic saturated saline, saturated sodium bicarbonate, and saturated saline, dried over anhydrous sodium sulfate, and the solvent was removed under reduced pressure to create the crude product, which was then recrystallized from dioxane/water to produce a yellow powder in a yield of 59.4%. ^1^H NMR (400 MHz, CDCl_3_) δ 8.62 (dd, *J* = 8.1, 1.4 Hz, 1H), 8.36 (d, *J* = 2.6 Hz, 1H), 7.70–7.55 (m, 3H), 7.55–7.40 (m, 2H), 6.48–6.34 (m, 1H), 5.81 (t, *J* = 1.6 Hz, 1H), 2.09 (t, *J* = 1.2 Hz, 3H).

Synthesis of PPI: TX-MMA, DMAEMA, TEAc-8, AIBN, and THF were added to a Shrek tube. After sufficient dissolution, oxygen in the system was removed by freeze-pump circulation, and the reaction was carried out at 70 °C for several hours according to different ratios and reaction conditions (Table 1). After the reaction is finished, it undergoes cooling to room temperature, the reaction solution is dropped into petroleum ether to precipitate, the precipitate is collected after centrifugation, washed with petroleum ether several times, and the product is collected by centrifugation and dried in a vacuum oven at 60 °C for 12 h to obtain the corresponding macromolecular PIs; molecular weights are summarized in Table 2.

### 2.4. Experimental Methods

Absorption spectrum: Prepare 5 × 10^−5^ mol/L PPI HPLC CH_3_CN solution. The optical path of the cuvette is 1 cm. According to the Beer–Lambert law (Equation (1)), the molar extinction coefficient of each wavelength band of PIs can be obtained.

A = εbc(1)

A is absorbance, ε is molar extinction coefficient, b is absorption layer thickness, unit/cm, c is concentration of absorbing substance, unit/mol/L.

Steady-state photolysis: Prepare 5 × 10^−5^ mol/L PPI HPLC CH_3_CN solution. At room temperature and air atmosphere, the steady-state photolysis experiments of the test samples were carried out with LED@365 nm and LED@405 nm; because PPI exhibits a broad absorption in the 250–430 nm range, 365 nm is close to the maximum absorption wavelength (λmax), while 405 nm is near the end of absorption. The irradiation power of the steady-state photolysis experiment is 5 mW/cm^2^.

Electron spin resonance (ESR): ESR experiments were measured with an EMXplus-10/12 X-band spectrometer. PPI-C (2 × 10^−2^ mol/L) and PBN (4 × 10^−2^ mol/L) were dissolved in toluene and deoxidized by argon for 15 min. Free radicals are generated after 15 min of LED@405 nm (100 mW/cm^2^) irradiation [31].

Photopolymerization kinetic: The kinetics of the polymerizations was studied in an RT-IR experiment. The PPI and monomer are mixed evenly to prepare the corresponding polymerization system. The polymerization system is coated on the brominated clavicle sheet, and the other side is covered with the brominated clavicle sheet to simulate the oxygen-free environment; the polymerization system is coated on the brominated clavicle sheet, and the other side is not covered with the brominated clavicle sheet to simulate the aerobic environment. RT-TR spectrometer (spectral range: 500–4000 cm^−1^, resolution 4 cm^−1^) was used to monitor the polymerization reactions. LED@365 nm and LED@405 nm were used for irradiation of the polymerization experiment; because PPI exhibits a broad absorption in the 250–430 nm range, 365 nm is close to the maximum absorption wavelength (λ_max_), while 405 nm is near the end of absorption, and the intensity was 80 mW/cm^2^. The polymerization kinetics were studied by monitoring the decrease in absorption peaks of the double bond (1650 cm^−1^) functional group to study the polymerization kinetics. The conversion is calculated using the following formula (Equation (2)):

Conversion (%) = [1 − A_0_/A_t_] × 100 (2)

A_t_ represents the characteristic peak area of the double bond at time t, and A_0_ represents the initial peak area.

Migration stability: A total of 1 wt% (TX-MMA content) of PIs was dissolved in 1 g of TMPTA monomer, dropped into a 15 mm diameter, 1.5 mm deep silica gel hole, and clamped with a cover glass. Soak the flakes in 10 mL of HPLC DCM at room temperature and perform UV-Vis absorption spectrum test at predetermined time (1 h, 2 h, 24 h, 36 h, 48 h). By calculating the numerical change in maximum absorbance, the mass of the extracted initiator can be calculated, and thus the migration rate of PIs can be calculated. The formula is shown as follows in Equation (3):

Relative fractional release (%) = (m_ex_/m_PI_) × 100 (3)

where m_ex_ refers to the mass of the extracted PIs and m_PI_ refers to the total mass of the PIs contained in the flakes.

## 3. Results

### 3.1. Photophysical and Photochemical Properties

**Figure 1:**
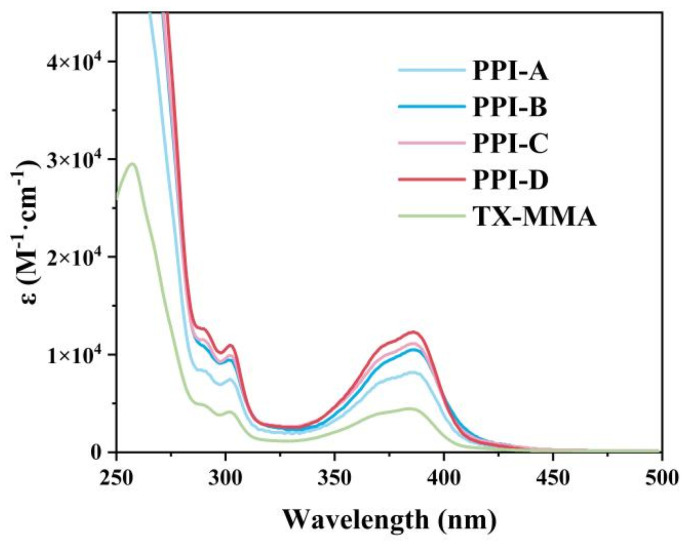
UV-Vis absorption spectra of PPI and TX-MMA in CH_3_CN at room temperature ([PI] = 5.0 × 10^−5^ mol/L).

As Figure 1 shows, the absorption of PPI (250–430 nm) can match the emission spectrum of LED lamps. The UV-Vis absorption spectra of PPI and the micromolecule TX-MMA have the same peak shape and no obvious change in the absorption wavelength, because covalent attachment of macromolecules does not change their conjugated structure. Notably, molar extinction coefficients of PPI are obviously higher than those of TX-MMA, which can be attributed to the existence of multiple chromophores in the macromolecules. In addition, the λ_max_ of PPI is 384 nm, and the other photophysical properties of PPI and TX-MMA are summarized in the Supplementary Materials (Table S1).

**Figure 2:**
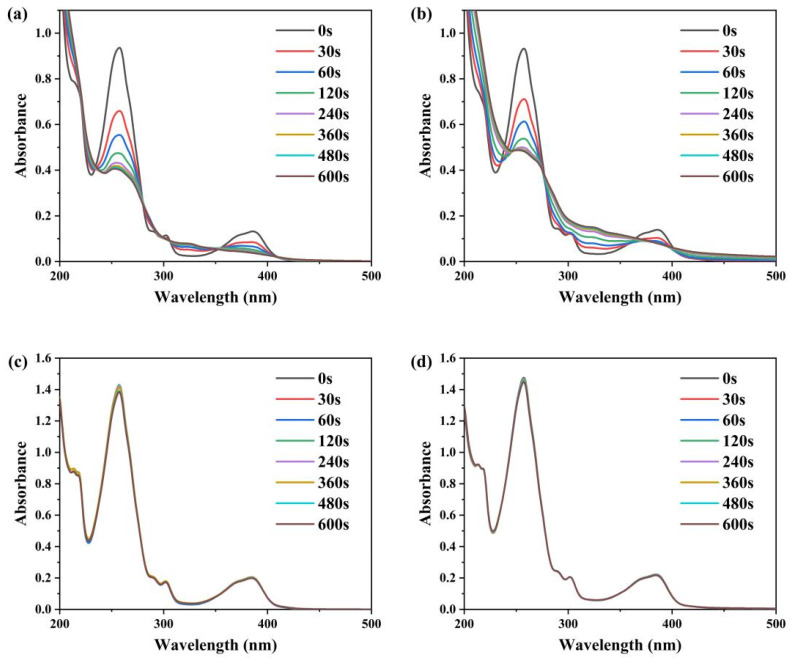
Steady-state photolysis curves of PPI-C and TX-MMA ([PI] = 5.0 × 10^−5^ mol/L) in CH_3_CN. PPI-C under LED@365 nm (**a**), LED@405 nm (**b**), and TX-MMA under LED@365 nm (**c**), LED@405 nm (**d**) irradiation.

The steady-state photolysis experiments of PPI and TX-MMA were carried out under the irradiation of LED@365 nm and 405 nm, and the results are shown in Figure 2. The absorption spectrum of TX-MMA does not change obviously with the increase in irradiation time. However, the obvious photolysis phenomenon can be observed in PPI, because TX-MMA is used as photoinitiation groups and N is used as a hydrogen donor; after irradiation, the excited TX-MMA and N undergo intramolecular or intermolecular hydrogen transfer reaction, and the thioxanthone skeleton is transformed into carbonyl radical. In addition, the photolysis rate under 365 nm light source irradiation is higher than 405 nm, perhaps because the molar extinction coefficient of PPI at 365 nm is higher than 405 nm, and the single photon energy of 365 nm is higher than 405 nm.

**Figure 3:**
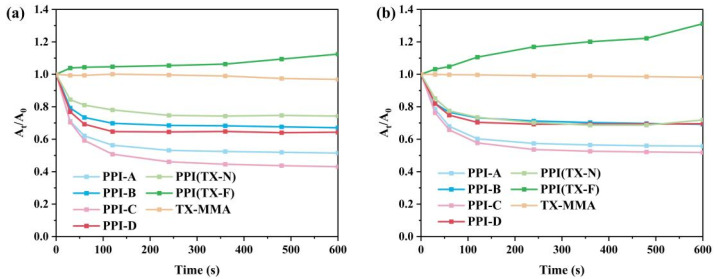
Plots of A_t_/A_0_ versus irradiation time for PPI under LED@365 nm (**a**) and LED@405 nm (**b**) irradiation.

The photolysis rates of the PPI and TX-MMA at λ_max_ = 384 nm (Figure 3) were evaluated by steady-state photolysis experiments, and the results are shown in Figure 3. Ternary PPI combines the reactivity of N unit and the migration of F unit, and the synergistic effect of the two results in ternary PPI having a higher photolysis rate. Compared to the other PIs, PPI-C exhibited the fastest photolysis, rate whether under LED@365 nm or 405 nm irradiation, which indicates that an increased ratio of N/F units to PPI, with an appropriate addition of thermal initiators and a prolonged reaction time, can obtain PPI with superior light absorption performance. Due to the migration ability of fluorine, ternary PPI diffuse more rapidly than PPI (TX-N) during photolysis, resulting in a faster decline rate of absorbance at 384 nm. The increased absorbance of PPI (TX-F) at 384 nm with prolonged irradiation is also attributed to the migration of fluorine, where PPIs from the underlying layer migrate to the upper layer, thereby enhancing the absorption value in the test area.

**Figure 4:**
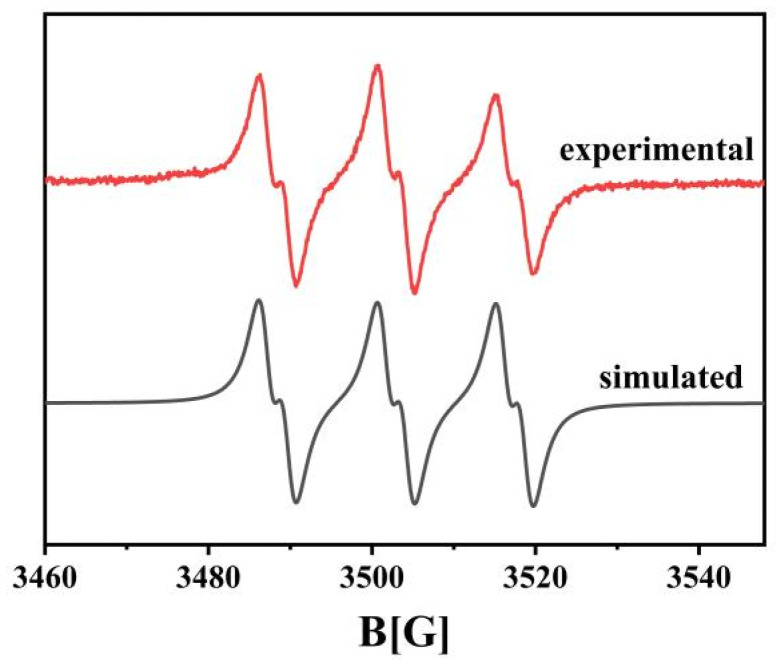
ESR spectra of PPI-C/PBN (2 × 10^−2^ mol L^−1^/4 × 10^−2^ mol L^−1^) experimental (red) and simulated (black).

**Figure 5:**
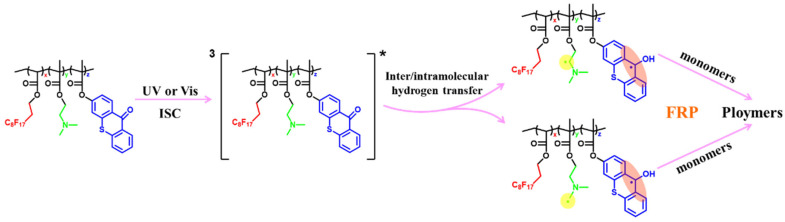
Proposed mechanism of photoinduced radical generation PPI.

In order to gain more insight into the photolysis mechanism, an ESR experiment was conducted. As shown in Figure 4, a solution of PPI-C was irradiated under LED@405 nm for 15 min, two carbon-centered radicals were generated, then captured by PBN, and the hyperfine coupling constants were a_N_ = 14.50 G and a_H_ = 2.51 G, which were in agreement with those observed for aminoalkyl radicals [25,32]. Based on the ESR experiment and previous studies [25], a possible initiation mechanism was proposed (Figure 5). PPIs reach the excited state after absorbing light, while aminoalkyl free radicals can be generated through intermolecular and intramolecular hydrogen transfer between carbonyl and aminoalkyl chains, thereby triggering polymerization. The hydrogen transfer reaction corresponds to an electron transfer, followed by a proton transfer process, and thus two sites are possible for the aminoalkyl radical.

### 3.2. Photopolymerization Kinetics

**Figure 6:**
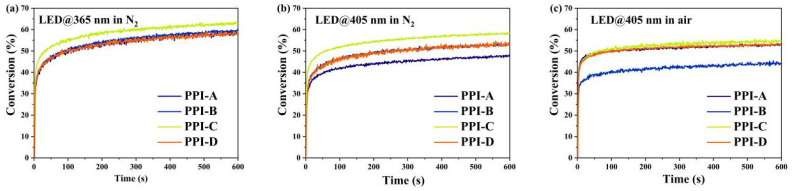
Kinetic profiles of TMPTA in the presence of PPI (5 wt%) under LED@365 nm (**a**), LED@405 nm (**b**) irradiation in N_2_, and under LED@405 nm (**c**) in air.

The initiation activity of PPI for free radical polymerization under irradiation of LED in air and nitrogen atmosphere was investigated by RT-IR. Kinetic profiles photoinitiated free radical polymerization of TMPTA under the irradiation of LED@365 and 405 nm, and they are presented in Figure 6. The results show that PPI-C has the best photoinitiation activity, whether under nitrogen or air condition, indicating that increasing the ratio of N/F unit to TX-MMA and prolonging the reaction time are helpful options to obtain PPI with lower oxygen inhibition.

In terms of specific analysis, PPI-C which increased the polymerization time from 6 h to 24 h has the best photoinitiation activity. Because the extended reaction time helps to improve the conversion of each unit, the higher the content of ternary macromolecules in PPI, the better the covalent connection of each unit, indicating that the structure of ternary macroinitiators helps to improve the activity of PPI. Compared to PPI-A, PPI-B only doubled the amount of TX-MMA used. Under LED@405 nm irradiation in nitrogen atmosphere, the final double bond conversion rate of PPI-B is 53% higher than that of PPI-A, which is 48%. However, under LED@405 nm irradiation in air, the final double bond conversion of PPI-B was 53%, while PPI-A decreased to 44%. At the same mass concentration, the higher the ratio of TX-MMA in PPI, the more active free radicals are generated to promote the polymerization reaction, while in air, there is an oxygen inhibition phenomenon, meaning a high ratio of reactive N and mobile F unit is more conducive to the reduction of oxygen inhibition. PPI-D, which decreased the addition of AIBN, has a higher conversion (Figure 6b) compared to PPI-A. Because of the wide molecular weight distribution of PPI (summarized in Table 2), the M_n_ changes little when reducing the addition of AIBN; however, the proportion of polymers with a large molecular weight is greater, which can be seen from GPC spectra (shown in the Supplementary Materials Figure S4), indicating that the decrease in AIBN is beneficial to the increase in polymerization degree, which is conducive to intermolecular hydrogen transfer. However, if the addition of AIBN is decreased continuously, the molecular weight of the polymer may be too large to dissolve because of the increased viscosity, which is not conducive to the photoinitiation efficiency of PPI. Therefore, the amount of AIBN added needs to be considered comprehensively.

**Figure 7:**
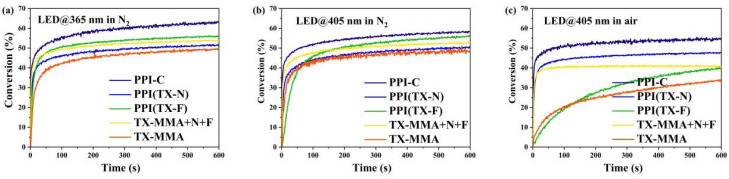
Kinetic profiles of TMPTA in the presence of PPI-C (5 wt%), TX-MMA (1 wt%), and TX-MMA+N+F (1:3:1 wt%) under LED@365 nm (**a**), LED@405 nm (**b**) irradiation in N_2_, and under LED@405 nm (**c**) in air.

The initiation efficiency of ternary/binary PPI and TX-MMA for photoinitiated free radical polymerization was also examined by using TMPTA (Figure 7). TX-MMA were mixed with N and F according to the proportion of units in PPI-C, which has the best photoinitiation activity. The ratio of each repeating unit in PPI-C was calculated by integrating the corresponding regions of different hydrogen atoms, which was judged by the ^1^H NMR spectrum of PPI-C given in the Supplementary Materials (Figure S3), comprising the ratio of x:y:z = 0.64:6.21:1, and the mass ratio of TX-MMA:N:F = 1:3:1. Under LED@405 nm irradiation, the final conversion of PPI-C in air decreased by only 3% compared to that in nitrogen atmosphere, while the final conversion of TX-MMA decreased by 15%, which demonstrates that the reactivity of N unit and the mobility of F unit in PPI-C decreased oxygen inhibition significantly. PPI-C has higher initiating activity than TX-MMA and N/F unit when added separately in nitrogen atmosphere or air, and it is also superior to binary PPI, which indicates that TX-MMA covalently linked with N and F unit is more conducive to improving the initiating efficiency of PPI. Compared to TX-MMA mixed with N and F units, the conversion of PPI-C was increased by 33.6% when irradiated with LED@405 nm in air.

### 3.3. Migration Stability

**Figure 8:**
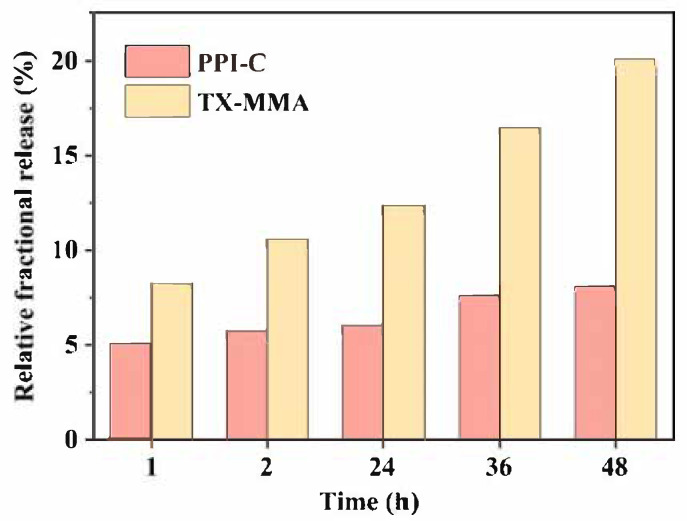
Plot of migration rate of PPI-C (5 wt%) and TX-MMA (1 wt%) cured flakes with time in DCM.

Migration stability of PIs is a significant parameter for applications in food and pharmaceutical packaging fields. In order to study the migration of PPI, the migration rates of PIs over time in TMPTA initiated by PPI-C (5 wt%) and micromolecule TX-MMA (1 wt%) were tested through the UV-Vis absorption spectrum of the leaching solution. DCM was chosen as the solvent because of its good solubility of TX and cleaved micromolecules. As shown in Figure 8, TX-MMA has high mobility due to its low molecular weight, and the migration rates of TX-MMA are 8.2% and 20.1% after extraction for 1 h and 48 h in DCM, while PPI has migration rates of 5.1% and 8.1%, respectively, which is 60% lower than TX-MMA. The results show that PPI’s macromolecular structure and high relative molecular mass overcome the low surface energy of F unit; thus, PPI still has lower migration and toxicity.

## 4. Conclusions

In summary, the one-component PPI was synthesized by utilizing a TX skeleton as the chromophore, and acrylate-containing tertiary amine and long fluorocarbon chain as the functional groups. PPI exhibits a broad absorption band spanning 250–430 nm, and stacking of multiple TX chromophores enhances the molar extinction coefficient. Under the irradiation of UV-Vis light, TX and N units in PPI generate aminoalkyl active radicals through intramolecular or intermolecular hydrogen transfer to initiate monomers curing. Utilizing the cooperative effect strategy of the reactivity of N unit and the migration of F unit efficiently reduced the oxygen inhibition in radical polymerization, and PPI exhibits excellent performance in initiating polymerization in air, while monomer conversion increased by 33.6%, which, compared to TX-MMA, mixed well with the N and F units. Moreover, increasing the N/F to TX ratio and prolonging the reaction time further reduced the oxygen inhibition of PIs. The macromolecular structure of PPI exhibits 60% lower migration compared to the micromolecule TX-MMA. PPI can migrate to the surface to reduce oxygen inhibition when initiating free radical polymerization, but it is not easy to migrate out of the surface after curing, thus reducing toxicity. It is important that our research results provide some guidance for designing TX PIs with a reduction of oxygen inhibition and migration and make the applications of photocuring materials in food-grade packaging and biomedical fields possible.

## Figures and Tables

**Table 1:** The materials used in this paper.

Name of Material	Manufacturer
Thiosalicylic acid	Adamas Reagents (Shanghai, China)
Dioxane	Adamas Reagents (Shanghai, China)
Methacryloyl chloride	Adamas Reagents (Shanghai, China)
Perfluorooctyl ethyl acrylates (TEAc-8)	Adamas Reagents (Shanghai, China)
N-tert-butyl-α-phenylnitrone (PBN)	Adamas Reagents (Shanghai, China)
Triethylamine	Sinopharm Chemical Reagent (Shanghai, China)
Acetonitrile (CH_3_CN)	Sinopharm Chemical Reagent (Shanghai, China)
Petroleum ether	Sinopharm Chemical Reagent (Shanghai, China)
Sodium chloride	Sinopharm Chemical Reagent (Shanghai, China)
Sodium bicarbonate	Sinopharm Chemical Reagent (Shanghai, China)
Anhydrous sodium sulfate	Sinopharm Chemical Reagent (Shanghai, China)
Toluene	Sinopharm Chemical Reagent (Shanghai, China)
Concentrated sulfuric acid	Sinopharm Chemical Reagent (Shanghai, China)
Phenol	Sinopharm Chemical Reagent (Shanghai, China)
Dimethylaminoethyl methacrylate (DMAEMA)	Sinopharm Chemical Reagent (Shanghai, China)
Azobisisobutyronitrile (AIBN)	Sinopharm Chemical Reagent (Shanghai, China)
Tetrahydrofuran (THF)	Sinopharm Chemical Reagent (Shanghai, China)
Dichloromethane (DCM)	Sinopharm Chemical Reagent (Shanghai, China)
Trimethylolpropane triacrylate (TMPTA)	Kailin Ruiyang Chemical (Changzhou, China)

**Table 2:** Mass of each reactant and polymerization time in the preparation of PPI.

PIs.	TX-MMA/g	DMAEMA/g	TEAc-8/g	AIBN/g	Time/h	M_n_/g/mol	M_w_/M_n_
PPI-A	1	3	1.5	0.12	6	1834	44.696
PPI-B	2	3	1.5	0.12	6	1173	33.066
PPI-C	1	3	1.5	0.12	24	1483	48.132
PPI-D	1	3	1.5	0.06	6	1767	49.204
PPI (TX-N)	1	3	0	0.12	24	1001	34.583
PPI (TX-F)	1	0	1.5	0.12	24	4994	2.935

## Data Availability

All data used during this study appear in the submitted article.

## Supplementary Materials

The following supporting information can be downloaded at: https://www.mdpi.com/article/10.3390/polym17162252/s1, Figure S1: 1H NMR spectrum of TX-OH; Figure S2: 1H NMR spectrum of TX-MMA; Figure S3: 1H NMR spectrum of PPI-C; Figure S4: GPC curves of the ternary and binary PPIs; Table S1: Photophysical properties of the investigated PPIs and TX-MMA.
