# Supplementary material for: Thioxanthone Skeleton-Based One-Component Macro-Photoinitiator Reduces Oxygen Inhibition and Migration Through Cooperative Effect
Source: Polymers (Basel). 2025 Aug 20;17(16):2252. doi: 10.3390/polym17162252 (PMC12390099; doi:10.3390/polym17162252)
Supplement: Supplementary file 1 [file polymers-17-02252-s001.zip › polymers-3818206-supplementary.pdf]

Supplementary Informantion

Thioxanthone Skeleton Based One Component Macro-Photoinitiator Reduces Oxygen Inhibition and Migration through Cooperative

Yiyun Du [1], Jingyan Zhang [1], Tianyi Han [3*] and Yi Zhu [1,2*]

1 International Research Center for Photoresponsive Molecules and Materials, Jiangnan University, 214122, Wuxi, Jiangsu (P. R. China).

2 Key Laboratory of Synthetic and Biological Colloids, Ministry of Education, School of Chemical and Material Engineering, Jiangnan University, 214122, Wuxi, Jiangsu (P. R. China).

3 State Key Laboratory of Tribology in Advanced Equipment, 100084, Beijing (P. R. China).

* Correspondence: zhuyi@jiangnan.edu.cn

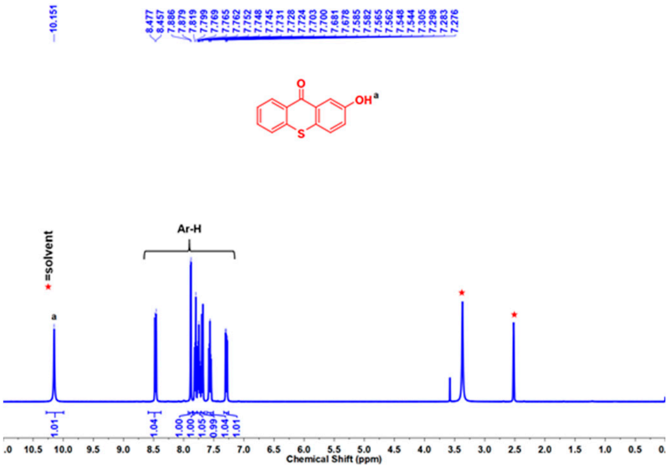

Figure S1. ^{1}H NMR spectrum of TX-OH

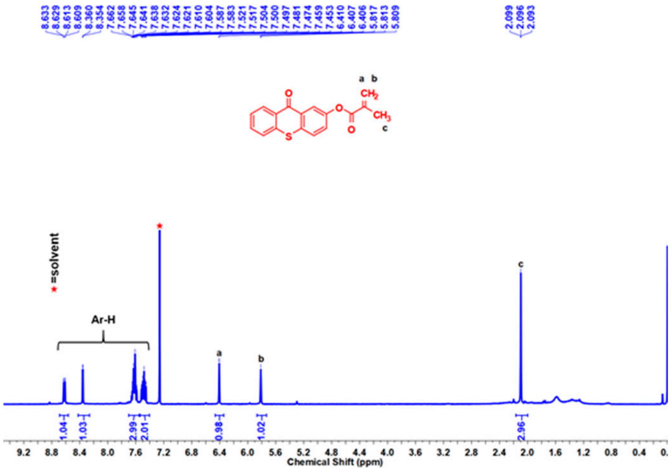

Figure S2. ^{1}H NMR spectrum of TX-MMA

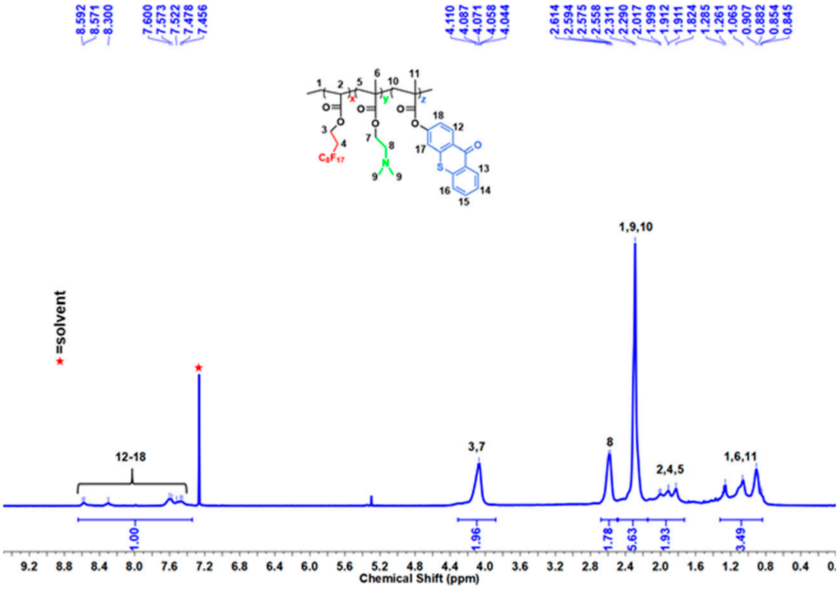

Figure S3. ^1H NMR spectrum of PPI-C

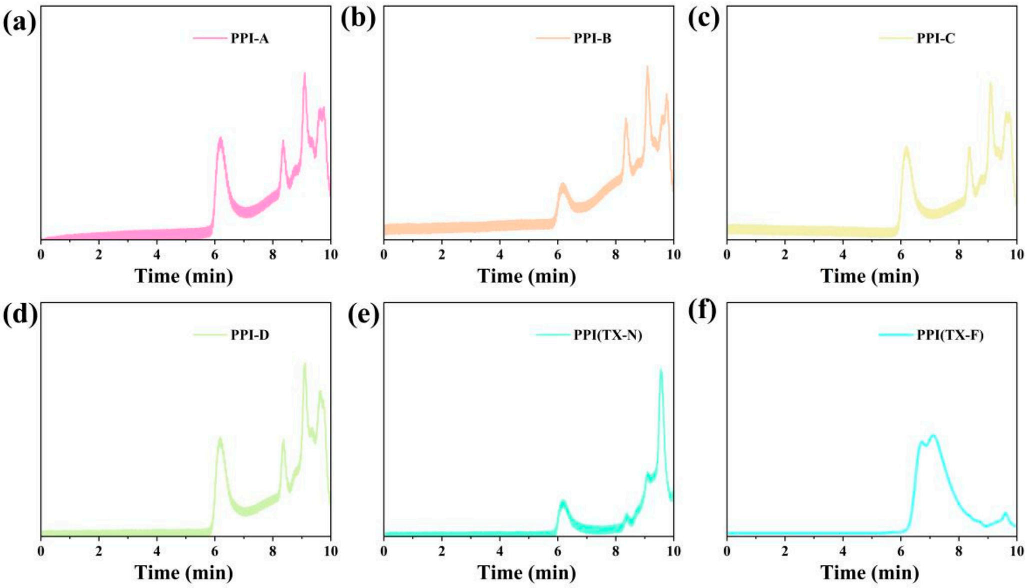

Figure S4. GPC curves of the ternary and binary PPIs

Table S1. Photophysical properties of the investigated PPIs and TX-MMA

PIs	λ_{max}/nm	$\varepsilon_{max}/10^3$ L mol^{-1} cm^{-1}	$\varepsilon_{365}/10^3$ L mol^{-1} cm^{-1}	$\varepsilon_{405}/10^3$ L mol^{-1} cm^{-1}
PPI-A	384	8.16	6.12	2.17
PPI-B	384	1.04	7.36	3.68
PPI-C	384	11.04	8.16	3.20
PPI-D	384	12.24	8.96	3.04
TX-MMA	384	4.46	3.54	0.92
